# Body Weight Perception and Eating Attitudes Among Polish Midwives with Overweight and Obesity: A Cross-Sectional Study

**DOI:** 10.3390/nu18010144

**Published:** 2026-01-01

**Authors:** Aleksandra Łopatkiewicz, Olga Barbarska, Iwona Kiersnowska, Beata Guzak, Edyta Krzych-Fałta

**Affiliations:** 1 Department of Nursing Propaedeutics, Faculty of Health Sciences, Medical University of Warsaw, 27 Erazma Ciolka St., 01-445 Warsaw, Poland; aleksandra.lopatkiewicz@wum.edu.pl (A.Ł.); edyta.krzych-falta@wum.edu.pl (E.K.-F.); 2 School of Medical & Health Sciences, VIZJA University, 59 Okopowa St., 01-043 Warsaw, Poland; 3 Department of Lifestyle Medicine, School of Public Health, Centre of Postgraduate Medical Education, 01-828 Warsaw, Poland; beata.guzak@cmkp.edu.pl

**Keywords:** midwives, body weight perception, overweight, obesity, eating attitudes, EAT-26, weight misperception

## Abstract

**Background:** Midwives, despite their health-promoting role, face factors that may disrupt eating behaviours and weight regulation. Little is known about their body weight perception or disordered eating attitudes (DEAs). This study assessed body weight perception and eating attitudes across BMI categories among Polish midwives. **Methods:** A cross-sectional survey of 568 midwives was conducted. BMI was calculated from self-reported measures and classified according to WHO criteria. Body weight perception was assessed using discrepancies between actual and ideal body weight and between self-perceived ideal body weight and ideal body weight. Long-term weight variability was additionally evaluated using the difference between maximum and minimum adult body weight. Eating attitudes were examined using the Polish version of the EAT-26. Group differences were analysed with the Kruskal–Wallis and χ^2^ tests. **Results:** Among the participants, 62.9% had normal weight, 23.4% were overweight, and 13.7% were obese. Perceived ideal body weight increased with BMI (*p* < 0.001). Midwives with overweight and obesity demonstrated higher EAT-26 scores than those with normal BMI, with EAT-26 > 20 observed in 8.3% of overweight and 14.1% of obese participants (*p* = 0.010). Overweight and obese midwives also showed larger discrepancies between actual and ideal body weight and greater lifetime weight variability, and these groups simultaneously presented higher levels of disturbed eating attitudes. Emotional eating, binge-type episodes, and dieting behaviours were more common among overweight and obese participants, while calorie awareness remained consistently high across groups. **Conclusions:** Midwives with excess body weight often misperceive their body size and show an elevated risk of DEA. Weight perception appears more strongly related to maladaptive eating patterns than BMI alone. These findings highlight the need for targeted, non-stigmatising interventions addressing weight perception, eating attitudes, and occupational stressors, which may support both midwives’ well-being and their professional effectiveness in delivering nutrition and lifestyle counselling.

## 1. Introduction

Overweight and obesity remain among the leading global public health challenges. According to the World Health Organization, one in eight people worldwide now lives with obesity, and over 2.5 billion adults are overweight [1]. High body mass index (BMI) is one of the fastest-rising metabolic risk factors, contributing substantially to global disability-adjusted life years (DALYs) and being implicated in more than 200 disease conditions [2].

In women, obesity is associated not only with cardiometabolic disorders but also with a range of reproductive and hormonal complications, including menstrual irregularities, endometrial and breast cancer, and menopausal symptoms [3]. These health consequences are particularly relevant for women of reproductive age and underscore the importance of weight-related health behaviours in professions centred on women’s healthcare.

Healthcare professionals—particularly nurses and midwives—are expected to model healthy lifestyles and promote preventive health behaviours. However, international data indicate a higher prevalence of overweight and obesity in these occupational groups compared with the general population [4,5]. Occupational factors such as shift work, irregular meals, disrupted sleep–wake rhythms, and chronic stress have been consistently identified as major contributors to unhealthy dietary patterns and weight gain in healthcare workers [6,7,8,9].

Exposure to chronic occupational stress may also increase vulnerability to maladaptive eating behaviours, including emotional eating, dietary restraint, and preoccupation with body weight. Studies among healthcare workers further suggest that psychological distress, weight stigma, and reduced well-being may negatively influence health-related coping mechanisms, even in individuals with professional knowledge of nutrition and disease prevention [10,11].

Despite extensive research on nurses, midwives remain a comparatively underexplored professional group. This gap is notable given that midwifery practice involves providing nutritional guidance and promoting healthy lifestyle behaviours among women across the reproductive life course [4]. Moreover, empirical data on weight perception and eating attitudes among midwives are scarce, particularly in Central and Eastern Europe, where cultural norms related to body image and professional roles may differ from those described in Western populations.

In Poland, midwifery is regulated as an autonomous medical profession with legally defined competencies, including independent management of physiological pregnancy, labour, postpartum care, and health education. Midwives complete a dedicated university-level programme and hold statutory responsibility for counselling on nutrition, weight control, and healthy lifestyle behaviours during pregnancy [12]. The combination of professional autonomy, a strong educational emphasis on prevention, and expectations related to health modelling may shape midwives’ own eating attitudes and perceptions of body weight.

At the same time, midwifery practice is characterised by high workload, emotional responsibility, frequent exposure to shift work, and unpredictable working hours, which are strongly associated with lifestyle disruptions [6,7]. A recent multinational systematic review indicates that midwives often experience emotional exhaustion, limited organisational support, and reduced autonomy, suggesting heightened vulnerability to psychological strain and maladaptive coping behaviours [13]. Such working conditions may contribute to irregular eating patterns, difficulties maintaining healthy habits, and distorted weight perception.

Eating attitudes and body weight perception represent important behavioural mechanisms linking occupational stress to health outcomes. Screening instruments such as the Eating Attitudes Test (EAT-26) enable early identification of disordered eating attitudes and related risk patterns [14,15]. Body weight misperception may further influence eating-related attitudes and weight-management behaviours [16,17]. Research in general female populations shows that women frequently misestimate both their own body size and the BMI category of “ideal” bodies, often shifting perceived ideals toward thinner silhouettes [18]. Similarly, studies among patients and healthcare professionals demonstrate substantial inaccuracies in recognising overweight and obesity [19]. Although these mechanisms have not been examined specifically in midwives, they may be particularly relevant given the high prevalence of occupational stress and weight-related health risks in this group.

Importantly, despite the extensive body of literature on body image and eating attitudes, existing research has largely focused on college-aged women and men [20,21,22], with comparatively limited attention paid to healthcare professionals. Midwives, irrespective of national context, occupy a uniquely sensitive clinical role that combines responsibility for women’s health promotion with high emotional and occupational demands. However, midwives’ own perceptions of body weight, eating attitudes, and related vulnerabilities remain largely overlooked in the literature.

Given these findings, this study aimed to assess body weight perception and eating attitudes among Polish midwives, with particular attention to differences across BMI categories and indicators of disordered eating. By focusing on this distinct professional group—whose working conditions markedly differ from those of nurses in many countries and whose education and occupational tasks are uniquely defined in Poland [12]—the research addresses a critical gap in the literature. Understanding weight perception and eating attitudes among midwives provides essential insight into the behavioural and psychological correlates of health risks in a profession central to women’s healthcare.

## 2. Materials and Methods

Participants were recruited using a convenience sampling strategy during continuing education courses and training events across Poland. A total of 568 professionally active midwives were included in the final analysis. Eligible participants were ≥18 years old, currently employed as midwives, and able to provide complete self-reported anthropometric data. Only individuals with normal, overweight, or obese BMI were included; participants with underweight were excluded due to their very small number and their distinct clinical and behavioural profile, which differs from mechanisms relevant to overweight and obesity—the primary focus of this study. Including this group could have reduced the clarity of BMI-category comparisons and introduced additional confounding.

As this was a non-interventional, anonymous questionnaire survey, it was notified to the Bioethics Committee at the Medical University of Warsaw (AK-BE/104/2025). The survey was administered in a paper-and-pencil format, and completion of the questionnaire was considered to constitute informed consent to participate in the study and for the collected data to be used for research purposes. The questionnaire was administered with standardised written instructions provided to all participants.

Anthropometric data and weight perception indices: Participants self-reported their height and weight, which were used to calculate body mass index (BMI, kg/m^2^). BMI categories were defined according to the World Health Organization (WHO) criteria: normal weight (18.5–24.9), overweight (25.0–29.9), and obesity (≥30.0).

Ideal body weight was calculated using the Lorentz formula (for women, ideal body weight [kg] = height [cm] − 100 − (height [cm] − 150)/2) [23], which is commonly applied as an anthropometric reference in European epidemiological research. Self-assessed ideal body weight was recorded as the participants’ self-reported desired body weight.

Weight perception indices were simplified into three measures:

(1) Deviation from ideal body weight, calculated as the difference between current body weight and ideal body weight estimated using the Lorentz formula;
(2) Deviation from self-assessed ideal body weight, defined as the difference between self-reported desired body weight and Lorentz-estimated ideal body weight;
(3) Lifetime weight variability, calculated as the difference between each participant’s highest and lowest adult body weight.

Together, these measures allowed assessment of the extent to which participants’ current and “desired” body weights aligned with anthropometric norms, as well as the magnitude of long-term weight fluctuations. Anthropometric measures were self-reported, which may be subject to reporting bias; however, this approach is widely used and considered acceptable in population-based epidemiological research.

Eating attitudes: Eating attitudes were assessed using the Polish version of the Eating Attitudes Test (EAT-26) [15]. The total EAT-26 score was used as the primary indicator of disordered eating attitudes and served as the main outcome measure in the analysis. A score above 20 was interpreted as reflecting a potential risk of disordered eating rather than a diagnostic status. In the present sample, the internal consistency of the scale was high (Cronbach’s α = 0.87). In addition to the total score, selected individual items (1, 4, 6, 11, 12, 17, and 23) were analysed a priori based on their clinical relevance for capturing specific dimensions of eating attitudes, including fear of weight gain, binge-eating tendencies, calorie awareness, and dieting behaviours, which may vary across BMI categories.

Age and years of professional experience were collected as descriptive variables. No multivariable adjustment for potential confounders was performed.

Statistical analysis: The data distribution was examined using the Shapiro–Wilk test. Due to the non-normality of the data distribution, non-parametric tests were used for the analysis (Kruskal–Wallis and χ^2^). Bonferroni correction was used within families of related tests, balancing Type I and Type II errors. The significance level was set at *p* < 0.017. All analyses were performed with Statistica software, version 13.3 (StatSoft, Kraków, Poland).

## 3. Results

This study included 568 midwives (mean age 33.1 ± 9.3 years; mean work experience 9.4 ± 8.6 years). The majority had a normal BMI (62.9%), while 23.4% were overweight, and 13.7% obese. Midwives with overweight and obesity were significantly older and had longer professional experience compared with those with normal BMI (*p* < 0.001). Work experience increased progressively across BMI categories (Table 1), with the highest median values observed in the obesity group. After controlling for BMI category, significant differences were observed across most EAT-26 items. Midwives with overweight and obesity reported stronger fear of weight gain, more frequent dieting behaviours, and greater preoccupation with body shape and calorie control than those with normal BMI (*p* < 0.001). Emotional eating and binge-type episodes were also more common in higher BMI groups. The only item without statistically significant differences was awareness of caloric content, which remained consistently high in all participants (median = 4; *p* = 0.155) (Table 1).

Indicators of body weight perception across BMI categories (Table 2) showed clear and statistically significant differences between the groups. Midwives with overweight and obesity exhibited greater deviations of their current body weight from the ideal value, as well as higher lifetime weight variability.

## 4. Discussion

This study provides novel evidence on body weight perception and eating behaviours among midwives in Poland, a professional group that has been largely overlooked in obesity research. Our results show that overweight and obesity were associated with substantial discrepancies between current, expected, and self-perceived ideal body weight, as well as with greater weight fluctuations over the life course. Higher BMI was consistently linked to elevated EAT-26 scores and more frequent episodes of weight cycling, with 6.4% of midwives with obesity reporting a history of treatment for eating disorders (*p* < 0.001).

These findings are partly consistent with results in young women, where ideal bodies outside the normal range (both underweight and overweight) were frequently misclassified as “normal” BMI [18]. Comparable misperceptions have been observed among Indian medical and nursing undergraduates, indicating that inaccurate body weight perception is also common among future healthcare professionals [20]. Overall, these findings suggest that workplace culture and professional norms may reinforce distorted perceptions of body weight. The internalisation of higher BMI as “acceptable” could represent a social adaptation, especially in occupational groups exposed to high stress and irregular work hours. Such mechanisms indicate that body image perceptions are shaped not only by individual factors but also by collective norms within healthcare teams. From a theoretical perspective, concepts derived from stress-coping models and social cognitive theory may help contextualise the observed relationships between occupational factors, eating attitudes, and body weight among midwives [24].

Analysis of individual EAT-26 items in our study revealed that the only area without significant differences across BMI categories was awareness of the caloric content of consumed foods. This finding indicates that self-reported caloric awareness was comparable across BMI groups and did not differentiate participants according to body weight status. Given the self-reported and attitudinal nature of this item, it likely reflects perceived awareness rather than actual dietary monitoring or effective caloric regulation. Consequently, similar scores across BMI categories should not be interpreted as evidence of equivalent dietary behaviours or metabolic outcomes. Similar discrepancies have been reported among Polish nurses, who frequently present with excess weight and metabolic risk factors despite professional education in disease prevention [25]. International evidence among nursing students further confirms that occupational fatigue, time constraints and work-related stress often impede the translation of knowledge into consistent healthy behaviours [21].

The results of the present study indicate a risk of disordered eating attitudes (DEAs) among midwives, with higher scores observed particularly in participants with overweight and obesity. As the EAT-26 is a screening instrument, these findings should be interpreted as reflecting subclinical risk patterns rather than diagnostic eating disorders. In the context of midwifery, DEA may not only compromise personal well-being but also influence professional functioning and credibility in providing lifestyle and nutrition counselling to pregnant women [26]. Evidence suggests that healthcare professionals’ own weight-related concerns may influence the quality and comfort of patient counselling [22], indicating that elevated eating-related concerns among healthcare professionals may affect not only their own health but also the quality of patient counselling.

Age and cumulative professional exposure may partly contextualise the observed weight-related patterns in this group. In our study, midwives with overweight and obesity were significantly older than those with normal BMI. Similarly, years of professional experience differed across BMI categories, with progressively longer work experience observed among midwives with overweight and obesity compared with those with normal BMI. Because age and years of professional experience are highly correlated in this population, these descriptive patterns cannot distinguish whether excess body weight is more closely associated with age, cumulative occupational exposure, or their joint influence. 

Work-related stressors, limited opportunities for self-care, and the unpredictable nature of midwifery work—such as interrupted breaks and emergency duties—may undermine regular eating patterns and promote reliance on less healthy food options [27].

These results also suggest that tailored preventive and educational initiatives, adapted to the specific demands of the midwifery profession, could support healthier eating behaviours and promote better weight regulation, ultimately enhancing midwives’ well-being and the quality of the health education they provide to pregnant women.

This study has several limitations. The EAT-26 serves as a screening rather than a diagnostic instrument, and the present analysis primarily emphasised the total score and selected items, which may not adequately reflect the full complexity of eating disorders. Moreover, certain potentially relevant environmental and psychosocial factors—such as detailed work schedules, the availability of healthy food options in the workplace, stress levels, and social support—were not comprehensively assessed, although they may have influenced body weight and dietary behaviours. The use of self-reported height and weight may have introduced reporting bias, particularly underestimation of body weight among participants with overweight and obesity; however, self-reported anthropometrics were appropriate given the study’s focus on body-weight perception. The cross-sectional design of the study also limits the ability to establish causal relationships between occupational factors, body weight, and eating attitudes.

An additional limitation is the exclusion of underweight participants from the analysis, which was dictated by the small size of this group and its distinct metabolic and psychological risk profile, potentially unrelated to the mechanisms leading to overweight and obesity among healthcare workers. Including this heterogeneous subgroup could distort the interpretation of BMI-related associations and reduce the methodological consistency of the analyses. Furthermore, the exploratory nature of the study means that the obtained results should be interpreted as descriptive and hypothesis-generating, requiring confirmation in future longitudinal studies using objective anthropometric measurements and a broader range of environmental and occupational variables.

Notwithstanding these limitations, the study offers valuable insights into body weight perception and eating behaviours among Polish midwives and highlights important directions for future research and preventive interventions. These findings highlight the need for tailored, non-stigmatising workplace interventions aimed at supporting midwives’ eating behaviours, stress management, and body-weight perception. Improving midwives’ well-being may also enhance their professional effectiveness in delivering nutrition and lifestyle counselling to women.

Future research should extend these findings using longitudinal designs to examine how occupational stress, shift work patterns, and cumulative professional exposure influence changes in body weight perception and eating attitudes over time among midwives. Additionally, intervention-based studies evaluating workplace-tailored nutritional, psychological, and stress-management programs are warranted to determine their effectiveness in improving both midwives’ well-being and their capacity to deliver lifestyle counselling.

## 5. Conclusions

Polish midwives with overweight and obesity displayed higher EAT-26 scores, more frequent emotional and dieting-related behaviours, and a noticeable upward shift in self-perceived ideal body weight. These findings indicate that distorted body-weight perception and subclinical disordered eating attitudes may co-occur in midwives with excess weight, despite comparable levels of self-reported nutritional awareness across BMI categories. Importantly, discrepancies between actual, ideal, and self-perceived body weight appeared to be more strongly associated with maladaptive eating patterns than BMI alone, underscoring the relevance of perceptual factors in assessing eating-related risk among healthcare professionals.

Given the exploratory and cross-sectional nature of this Brief Report, the results should be interpreted as descriptive and hypothesis-generating rather than causal. Nevertheless, the observed patterns suggest that midwives may represent a professional group particularly vulnerable to weight-related psychological and behavioural risks, potentially shaped by cumulative occupational stress and working conditions inherent to midwifery practice.

These findings highlight the potential value of workplace-tailored, non-stigmatising preventive strategies that integrate nutritional education, psychological support, and stress-management resources within midwifery settings. Supporting midwives’ well-being may not only improve their own health but also enhance their professional effectiveness in delivering nutrition and lifestyle counselling to women.

## Figures and Tables

**Table 1:** Characteristics and selected behavioural indicators, including selected EAT-26 score ranges, according to BMI category (n = 568).

Variable	Normal BMI (n = 357)	Overweight (n = 133)	Obesity (n = 78)	*p*-Value
Age (years), median (IQR)	29 (25–37)	36 (29–43)	41 (34–46)	<0.001 *
Work experience (years), median (IQR)	6 (2–12)	11 (5–19)	15 (9–23)	<0.001 *
Self-perceived “ideal” body weight (kg), mean ± SD	57.6 ± 5.4	65.2 ± 6.3	68.7 ± 7.2	<0.001 *
EAT-26 > 20, n (%)	17 (4.8%)	11 (8.3%)	11 (14.1%)	0.010 *
Weight loss >10 kg in past 6 months, n (%)	9 (2.5%)	11 (8.3%)	10 (12.8%)	<0.001 *
Previously treated for eating disorder, n (%)	3 (0.8%)	2 (1.5%)	5 (6.4%)	<0.001 *
Reports emotional eating, n (%)	79 (22.1%)	45 (33.8%)	38 (48.7%)	<0.001 *
EAT-26 total score, median (IQR)	5 (0–32)	7 (0–50)	8 (0–31)	<0.001 *
“Am terrified about being overweight”, median (IQR)	3 (1–6)	4 (1–6)	4 (1–6)	0.008 *
“Have gone on eating binges where I felt unable to stop”, median (IQR)	2 (1–6)	2 (1–6)	3 (1–6)	<0.001 *
“Aware of the calorie content of the foods I eat”, median (IQR)	4 (1–6)	4 (1–6)	4 (2–6)	0.155
“Am pre-occupied with the desire to be thinner”, median (IQR)	2 (1–6)	3 (1–6)	3 (1–6)	<0.001 *
“Think about burning up calories when I exercise”, median (IQR)	3 (1–6)	3 (1–6)	3 (1–6)	0.002 *
“Eat diet foods”, median (IQR)	3 (1–6)	3 (1–5)	3 (1–6)	0.012 *
“Engage in dieting behavior”, median (IQR)	3 (1–6)	3 (1–6)	4 (1–6)	<0.001 *

* Statistical significance after Bonferroni correction: *p* < 0.017.

**Table 2:** Indicators of body weight perception according to BMI category (n = 568).

Indicators of Body Weight Perception	Normal BMI (n = 357)	Overweight (n = 133)	Obesity (n = 78)	*p*-Value
Difference between current body weight and ideal body weight (kg), mean ± SD	2.9 ± 5.1	16.7 ± 4.6	35.7 ± 12.4	<0.001 *
Difference between self-perceived ideal body weight and ideal body weight (kg), mean ± SD	−0.7 ± 3.7	6.6 ± 4.5	10.5 ± 6.2	<0.001 *
Difference between min and max body weight (kg), mean ± SD	11.2 ± 6.4	20.8 ± 9.3	29.5 ± 11.4	<0.001 *

* Statistical significance after Bonferroni correction: *p* < 0.017.

## Data Availability

The data underlying this article are available from the corresponding author upon reasonable request.

## Abbreviations

BMI	Body Mass Index
DALYs	Disability-Adjusted Life Years
DEA	Disordered Eating Attitude
EAT-26	Eating Attitudes Test (26 items)
WHO	World Health Organization
